# Functional inclusion bodies produced in the yeast *Pichia pastoris*

**DOI:** 10.1186/s12934-016-0565-9

**Published:** 2016-10-01

**Authors:** Fabián Rueda, Brigitte Gasser, Alejandro Sánchez-Chardi, Mònica Roldán, Sandra Villegas, Verena Puxbaum, Neus Ferrer-Miralles, Ugutz Unzueta, Esther Vázquez, Elena Garcia-Fruitós, Diethard Mattanovich, Antonio Villaverde

**Affiliations:** 1 Institut de Biotecnologia i de Biomedicina, Universitat Autònoma de Barcelona, Bellaterra, 08193 Cerdanyola del Vallès, Spain; 2 Departament de Genètica i de Microbiologia, Universitat Autònoma de Barcelona, Bellaterra, 08193 Cerdanyola del Vallès, Spain; 3 CIBER de Bioingeniería, Biomateriales y Nanomedicina (CIBER-BBN), Bellaterra, 08193 Cerdanyola del Vallès, Spain; 4 Department of Biotechnology, University of Natural Resources and Life Sciences Vienna (BOKU), Muthgasse 18, 1190 Vienna, Austria; 5 Austrian Centre of Industrial Biotechnology (ACIB), Muthgasse 11, 1190 Vienna, Austria; 6 Servei de Microscòpia, Universitat Autònoma de Barcelona, Bellaterra, 08193 Cerdanyola del Vallès, Spain; 7 Departament de Bioquímica i Biologia Molecular, Universitat Autònoma de Barcelona, Bellaterra, 08193 Cerdanyola del Vallès, Spain; 8 Department of Ruminant Production, Institut de Recerca i Tecnologia Agroalimentàries (IRTA), 08140 Caldes de Montbui, Spain; 9 Oncogenesis and Antitumor Drug Group, Biomedical Research Institute Sant Pau (IIB-Sant Pau), Hospital de la Santa Creu i Sant Pau, C/Sant Antoni Maria Claret, 167, 08025 Barcelona, Spain

**Keywords:** Recombinant proteins, Inclusion bodies, *Pichia pastoris*, Functional materials

## Abstract

**Background:**

Bacterial inclusion bodies (IBs) are non-toxic protein aggregates commonly produced in recombinant bacteria. They are formed by a mixture of highly stable amyloid-like fibrils and releasable protein species with a significant extent of secondary structure, and are often functional. As nano structured materials, they are gaining biomedical interest because of the combination of submicron size, mechanical stability and biological activity, together with their ability to interact with mammalian cell membranes for subsequent cell penetration in absence of toxicity. Since essentially any protein species can be obtained as IBs, these entities, as well as related protein clusters (e.g., aggresomes), are being explored in biocatalysis and in biomedicine as mechanically stable sources of functional protein. One of the major bottlenecks for uses of IBs in biological interfaces is their potential contamination with endotoxins from producing bacteria.

**Results:**

To overcome this hurdle, we have explored here the controlled production of functional IBs in the yeast *Pichia pastoris* (*Komagataella* spp.), an endotoxin-free host system for recombinant protein production, and determined the main physicochemical and biological traits of these materials. Quantitative and qualitative approaches clearly indicate the formation of IBs inside yeast, similar in morphology, size and biological activity to those produced in *E. coli,* that once purified, interact with mammalian cell membranes and penetrate cultured mammalian cells in absence of toxicity.

**Conclusions:**

Structurally and functionally similar from those produced in *E. coli*, the controlled production of IBs in *P. pastoris* demonstrates that yeasts can be used as convenient platforms for the biological fabrication of self-organizing protein materials in absence of potential endotoxin contamination and with additional advantages regarding, among others, post-translational modifications often required for protein functionality.

**Electronic supplementary material:**

The online version of this article (doi:10.1186/s12934-016-0565-9) contains supplementary material, which is available to authorized users.

## Background

Inclusion bodies (IBs) are insoluble protein clusters formed in *Escherichia coli* during recombinant protein production in the context of conformational stress [1]. Although protein aggregation is an obstacle for the production of soluble proteins, the biological activity shown by many IB-forming proteins represents an unexpected added value, of interest in the design of functional soft materials for different biotechnological and biomedical applications [2]. As an example, IBs formed by enzymes display catalytic activities similar to those of the enzyme in soluble form [3, 4]. In such immobilized version, enzyme-based IBs (and the analogous mammalian aggresomes) can be used in catalysis, maintaining their activity in several batch cycles of the reaction process [5]. In addition, the unusual combination of mechanical stability [6] (provided by amyloid-like fibrils, [7, 8]), membrane-crossing activities [9] and the ability to release functional protein upon cellular uptake [10] make IBs appealing substrate topologies and protein-releasing materials in both tissue engineering [11] and protein replacement therapies [10]. The above examples underscore usefulness of IBs and make them an attractive object of study with high potential for their application in biomedicine and related fields.

A major obstacle in the development of IBs as materials with biomedical applications is the presence of endotoxins in Gram-negative bacteria that must be cleared up before application in biological interfaces. While the removal of lipopolysaccharide (LPS) has been implemented for soluble protein products [12–14], it is not yet established for more complex materials such as IBs. As an alternative to endotoxin removal, IBs might be produced in endotoxin-free cell factories, a concept that has been already explored in finely engineered *E. coli* mutants but not in other common cell factories [15, 16]. In this context, the yeast *Pichia pastoris* (*Komagataella* spp.) is not only an endotoxin-free cell factory but also one of the most important microorganisms for recombinant protein production, both for soluble proteins but also as for complex oligomeric ensembles [17–19]. Among others, self-assembling peptides [20, 21], heterologous prion fibrils [22], oligomeric antigenic constructs [23] and conventional virus-like particles (VLPs) [24] have been successfully produced in this biological platform. In this context, and although conformational stress has been deeply examined in recombinant yeast [25–29], there are no studies reporting the controlled production of biologically active IBs or IB-like materials so far, and only a limited number of olden reports described clustered accumulation of non-functional recombinant protein in this system [30–35]. The controlled production of functional IBs in yeasts would provide an additional and highly appealing value. The composition of IBs, an issue not fully determined in the regular producer *E. coli*, might be largely variable and affected by process conditions and the particular protein. Since many yeast species have been formerly classified as generally recognized as safe (GRAS) [36] and suitable for the production of GRAS or food- or pharma-grade products [17, 19], IBs from yeasts would surely be biologically safer in in vivo administration (e.g., though oral delivery, [10]) than those produced in bacteria. Thus, we set out to examine the potential formation of IBs in *P. pastoris* as model yeast, by producing, intracellularly, an aggregation-prone GFP fusion protein (VP1GFP) that forms fully fluorescent IBs in *E. coli*. Since fluorescent, insoluble protein clusters rich in cross-molecular beta-sheet architecture are indeed formed upon recombinant gene expression, the data presented here clearly demonstrate that *P. pastoris*, as a representative yeast species, can be used as a convenient factory for the desired production of biologically active IBs.

## Methods

### Strains and plasmids

The *VP1GFP* gene sequence was codon-optimized for expression in *P. pastoris* and provided by GeneArt (Thermo Fisher Scientific, Waltham, MA, USA). An optimized gene containing an *Mfe*I restriction site at 5′ and *Sfi*I at 3′ (Additional file 1) was inserted into a constitutive pPM2dZ30-pGAP plasmid with the glyceraldehyde-3-phosphate dehydrogenase (GAP) promoter and Zeocin resistance as selection marker. Cloning was performed first using *E. coli* DH10B grown in Lysogeny Broth (LB) rich media with 25 µg mL^−1^ Zeocin, and plasmids were extracted and purified through a miniprep kit (Süd-Laborbedarf Gauting, Germany). Before transformation into *P. pastoris*, plasmids were linearized with *Avr*II restriction enzyme in the GAP promoter region for subsequent genome integration. Electrocompetent wild type *P. pastoris* strain CBS7435 (= *K. phaffii*) cells were transformed by electroporation (2000 V, 25 µF and 200 Ω) with 2.5 µg of the linearized plasmid, and plated on YPD-agar (per liter: 10 g yeast extract, 20 g soy peptone, 20 g glucose, 10 g agar–agar, pH set to 7.5 with NaOH) containing 50 µg mL^−1^ Zeocin and cultivated for 48 h at 28 °C. Ten positive transformants were selected and re-plated on YPD-agar On the other hand, plasmids and media as well as the production and purification of VP1GFP IBs from *E. coli* MC4100 were described in a previous study [37].

### Screening of transformants

Selected *P. pastoris* clones were pre-cultured in 24 deep well cultivation plates with selective YPD medium (2 mL per well) overnight at 28 °C and 180 rpm. 2 mL of fresh BM medium (per liter: 20 g glucose monohydrate, 10 g yeast extract, 10 g peptone, 100 mM potassium phosphate buffer pH 6.0, 13.4 g yeast nitrogen base with ammonium sulfate, 0.4 mg biotin) were then inoculated to an initial OD_600_ = 0.1, Glucose (1 %) was added every 12 h during cultivation at 28 °C and 180 rpm. Later, cultures were diluted to an OD_600_ = 0.5 and fluorescence emission at 510 nm (excitation at 450 nm) was measured in a 96 well plate reader (Infinity m200, Tecan, Männedorf, Switzerland). Two clones per construct with high (HY) and low (LY) fluorescence emission/protein yield were selected.

### Fluorescence microscopy

After cultivation in BM medium for 48 h, 1 mL of each culture was diluted to OD_600_ = 10. Samples were centrifuged at 3000×*g* and resuspended in PBS and 5 µL of the suspension were pipetted on a glass slide, covered with a cover slip and viewed in a Leica DMI 6000 fluorescence microscope using an HCX PL APO CS 100 × 1.4 NA oil-immersion objective, differential interference contrast (DIC) and a Leica L5 filter for GFP fluorescence (Leica Microsystems, Germany). Images were analyzed using LAS AF lite software (Leica Microsystems), and Image J software.

### IB production and purification

Isolated HY and LY clones were pre-cultured in 10 mL of YPD selective medium at 28 °C and 180 rpm overnight. 500 mL of BM medium in 2 L flasks were inoculated with the pre-cultures to an initial OD_600_ = 0.1 and were fed with 1 % glucose every 12 h during 48 h. Cultures were centrifuged at 5000×*g* for 15 min and pellets were resuspended in 25 mL of PBS (sterile and filtered). The new suspensions were again centrifuged and pellets were stored at −80 °C. Afterwards, pellets were diluted in 20 mM Tris-buffer pH 8.0 containing 0.5 M NaCl and yeast cells were disrupted by pressuring twice at 40 Kpsi below 40 °C, using a cellular disruptor TS-5 4KW (Constant Systems, Daventry Northants, UK). Disrupted samples were centrifuged and resuspended in 200 mL of PBS containing 0.4 mM phenylmethylsulfonylfluoride (PMSF) and EDTA-free protease inhibitor cocktail (Roche Diagnostics, Indianapolis, IN, USA). Mixtures were frozen and thawed several times until there was no evidence of bacterial contamination upon plating 100 μL of sample on LB-agar. Then, 100 μL of nonylphenoxypolyethoxylethanol (NP-40) (Roche Diagnostics) were added and samples incubated at 4 °C for 1 h. Later, 1 mM MgSO_4_ and DNase (1 μg/mL) were added and samples incubated at 37 °C for 1 h with shaking at 250 rpm and centrifuged at 15,000×*g* for 15 min at 4 °C. Pellets were resuspended in 25 mL of lysis buffer (50 mM TrisHCl, pH 8.0, 100 mM NaCl, 1 mM EDTA) containing 0.5 % Triton X-100 and 100 μL of each sample were plated again in LB agar as a contamination test. The samples were centrifuged at 15,000×*g* for 15 min at 4 °C and pellets resuspended in PBS and divided in 5 mL aliquots. All of the aliquots were centrifuged again at the same conditions as above and pellets were finally stored at −80 °C until use.

### Western blot

Disrupted samples were centrifuged at 7000×*g* for 30 min at 4 °C and supernatant (soluble cell fraction) were separated from pellet (insoluble cell fraction). Pellets were resuspended in 500 ml of 20 mM Tris pH = 8.0 and 0.5 M NaCl, containing EDTA-free protease inhibitor cocktail (Roche Diagnostics, Indianapolis, USA). Samples were then heated at 98 °C (10 min for the soluble fraction and 45 min for the insoluble fraction). 20 µL of both soluble and insoluble fraction were loaded separately into 10 % polyacrylamide gels for SDS-PAGE according to Laemmli’s method. After running, proteins were transferred to a nitrocellulose membrane (GE Healthcare, Buckinghamshire, UK) at 100 V 60 min using 0.2 M glycine, 25 mM Tris, and 20 % methanol (v/v) as a transfer buffer. The membrane was blocked with a 5 % milk powder-PBS blocking buffer and incubated 2 h with anti-GFP diluted 1/500 (sc-8334, Santa Cruz Biotechnology, Santa Cruz, CA, USA) as primary antibody following of 1 h incubation with HRP conjugated anti-rabbit IgG (H+L) antibody (Bio-Rad, Hercules, CA, USA) diluted 1/1000 as secondary antibody. Bands were developed with a SuperSignal Chemiluminescent Kit (Thermo Scientific) and visualized in a VersaDoc MP imaging system (Bio-Rad).

### Fluorescence and Attenuated Total Reflectance spectroscopy

IB pellets were resuspended in 1 mL of PBS and fluorescence was measured in a Cary Eclipse fluorescence spectrometer (Agilent Technologies, Mulgrave, Australia) at 25 °C. The measurements were performed at a excitation wavelength of 450 nm and emission of 510 nm.

Spectra were acquired at room temperature on a Variant Resolutions Pro spectrometer coupled to an Attenuated Total Reflectance (ATR) accessory. Pellets were resuspended in D_2_O, placed in the crystal surface (10 µL) and nitrogen gas dried. Two hundred fifty spectra, recorded at a scan rate of 95 cm^−1^/min and a nominal resolution of 2 cm^−1^, were averaged for each sample. The obtained series were corrected against a background and the buffer was subtracted (D_2_O). Data treatment and band deconvolution of the original amide I band were performed by using the GRAMS software (Thermo Scientific). Deconvolution was performed into a Lorentzian curve, using a band shape of 20 and a K factor in the bessel apodization of 2. The fitting of the bands to the original spectra was performed by setting the band shape to a Gaussian curve. The fitting was obtained by iteration in two steps: first, the band positions were fixed and then were left floating.

### Internalization trials

HeLa cells (ATCC reference CCL-2) were cultured into a treated 24 well plate (6 × 10^4^ cells per well) with Minimum Essential Medium (MEM, Gibco, Thermo Scientific) supplemented with 10 % FBS and 2 mM Glutamax (MEMα-FBS-G) 24 h at 37 °C and 5 % CO_2._ After 24 h, medium was removed and cells were immediately washed twice with DPBS. Afterwards, 5 µg of VP1GFP IBs along with Optipro medium (Gibco, Thermo Scientific) supplemented with 2 mM l-Glutamine were added. The plate was incubated 24 h at 37 °C and after incubation, 250 µL of trypsin (1 mg/mL) were added for 15 min to detach cells and to remove IB protein that might be externally associated. Trypsin was inactivated by the addition of 750 µL of MEMα-FBS-G and the cells were centrifuged at 1200×*g,* at 4 °C for 5 min. Obtained samples were analyzed on a FACS Canto system (Becton–Dickinson, Franklin Lakes, NJ, USA) using a 15 W argon-ion laser at 488 nm excitation and fluorescence emission was measured with a 530/30 nm band pass filter.

### Electron microscopy

Microdrops of VP1GFP IBs were deposited during 2 min in silicon wafers (Ted Pella Inc., Redding, CA, USA), air-dried and IBs morphometry (size and shape) at a nearly native state was studied with a field emission scanning electron microscope (FESEM) Zeiss Merlin (Oberkochen, Germany) operating at 1 kV and equipped with a high resolution in-*lens* secondary electron detector.

Pellets of VP1GFP IBs and *P. pastoris* cells were fixed in 4 % (w/v) paraformaldehyde (TAAB Lab., Reading, Berkshire, UK) and 0.1 % (v/v) glutaraldehyde (EM grade, Merck, Darmstadt, Germany) in phosphate buffer 0.1 M (Sigma-Aldrich, Steinheim, Germany), cryoprotected in glycerol (Sigma-Aldrich), cryofixed in propane in a EMCPC (Leica Microsystems, Wetzlar, Germany), dehydrated in methanol (Merck), and embedded in Lowicryl HM20 resin (Polysciences Inc., Warrington, PA, USA) in a EMAFS automatic freeze substitution system (Leica Microsystems). Ultrathin sections of selected areas were placed on carbon-coated gold grids and labelled following standard procedures for transmission electron microscopy (TEM). Briefly, sections were placed in blocking buffer BSA-PBS containing glycine, incubated overnight at 4 °C with a rabbit polyclonal anti-GFP diluted 1/25 (ab-6556, Abcam, Cambridge, UK) as primary antibody followed by 40 min incubation with protein A coupled to 10 nm-gold particles diluted 1/50 (BBI Solutions, Cardiff, Wales, UK). Grids were contrasted and examined with a TEM JEM-1400 (Jeol Ltd., Tokyo, Japan) equipped with a CCD Gatan ES1000 W Erlangshen camera.

### Confocal microscopy

HeLa cells were cultured as described above, re-plated into MatTek dishes (MatTek Corporation, Ashland, MA, USA) at 1.5 × 10^5^ cells per plate and incubated 24 h at 37 °C. Then, medium was removed and cells on the plates were carefully washed twice with DPBS. 5 µg of insoluble VP1GFP material were resuspended in 1 mL of Optipro medium supplemented with 2 mM l-Glutamine and added to plates for cell exposure, for 24 h at 37 °C. Plasma membrane was labeled with 5 µg/mL Cell Mask™ Deep Red (Life Technologies, Carlsbad, CA, USA) and DNA with 5 µg/mL Hoechst 33342 (Life Technologies) in darkness for 10 min.

The confocal images were collected on an inverted TCS SP5 Leica Spectral confocal microscope using a HCX PL APO lambda blue 63x/1.4 oil immersion objective. Image size was set to 1024 × 1024 pixels. Excitation was via a 405 nm blue diode laser, 488 nm line of an argon ion laser and 633 nm helium–neon laser. Optimized emission detection bandwidths were configured at 425–485 nm (Hoechst), 500–550 nm (GFP) and 650–800 nm (Cell Mask) by hybrid detector. Sequential acquisition settings were used to avoid inter-channel cross-talk. The confocal pinhole was set to 1 Airy unit. Z-stack acquisition intervals were selected to satisfy Nyquist sampling criteria. Confocal Z-stacks comprising up to 50 images were deconvolved using Huygens Essential software version 4.4.0 p6 (SVI, Leiden, The Netherlands) and were reconstructed into 3D projections with the aid of Imaris ×64 v. 7.2.1. software (Bitplane; Zürich, Switzerland) with Surpass mode.

### Statistical analysis

All data were analyzed by ANOVA procedures and significant differences between soluble and insoluble yield of VP1GFP protein in *P. pastoris* were established by paired T test. In addition, specific fluorescence between *E. coli* and *P. pastoris* IBs were compared by a Tukey HSD statistical test. Raw data for all the experiments is provided in the Additional file 2.

## Results

**Fig. 1:**
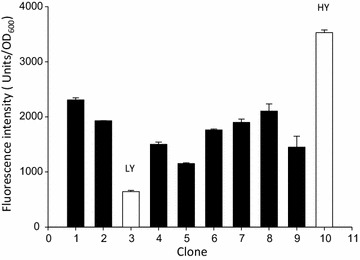
Fluorescence screening of *P. pastoris* clones producing VP1GFP. Clone # 3 was selected as low yield producer (LY) while # 10 as high yield producer (HY)

**Fig. 2:**
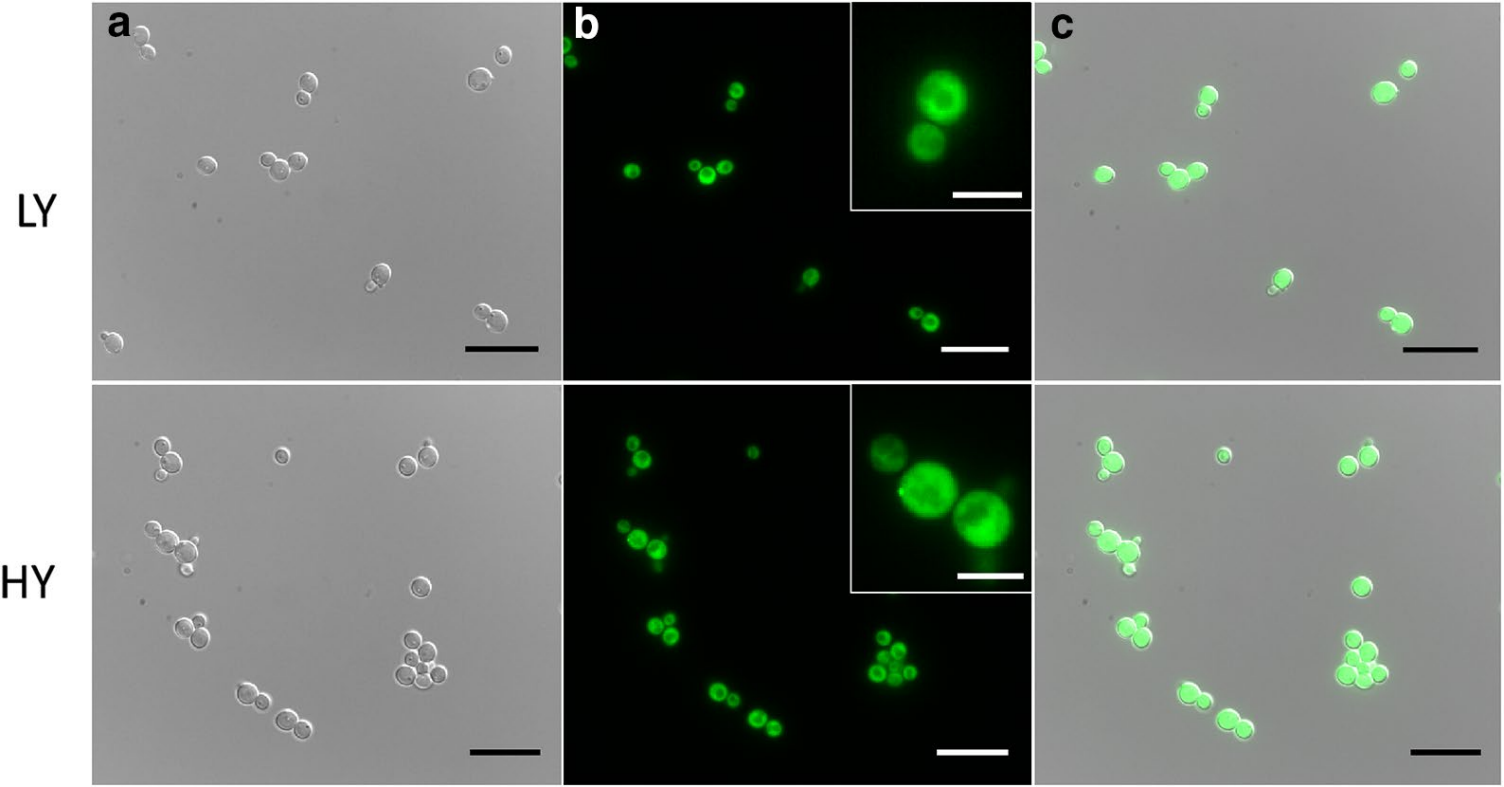
Imaging of LY and HY clones of *P. pastoris* producing VP1GFP. **a** DIC images at 100× magnification (*scale bar*, 20 µm). **b** Fluorescence images at 100× magnification (*scale bar*, 20 µm) with upper right insets (*scale bar*, 5 µm). **c** Merged images using LAS AF Lite Leica Software. Images might be not fully representative of actual fluorescence intensity, which is quantitatively reported in Fig. 1

*Pichia pastoris* cells were successfully transformed with the plasmid pPM2dZ30-pGAP encoding a VP1GFP version with a yeast-optimized gene sequence (Additional file 1). The fusion protein VP1GFP forms fluorescent IBs when overproduced in *E. coli* [3], because of the aggregation-prone nature of the VP1 moiety (derived from a foot-and-mouth disease virus VP1 protein). Ten *P. pastoris* transformants were cultivated in deep well plates and screened for their fluorescence. HY and LY clones were selected as representative of higher and lower producers within the set, respectively (Fig. 1), to evaluate the potential impact of gene dosage on the aggregation of encoded proteins and possible IB formation. LY and HY clones were checked by epifluorescence microscopy (Fig. 2). In both of them, the cytoplasm of individual cells appeared as fluorescent with heterogeneous areas of emission (Fig. 2b), an observation that might be compatible with protein deposition as aggregates. Essentially, all observed individual cells were fluorescent (Fig. 2c).

**Fig. 3:**
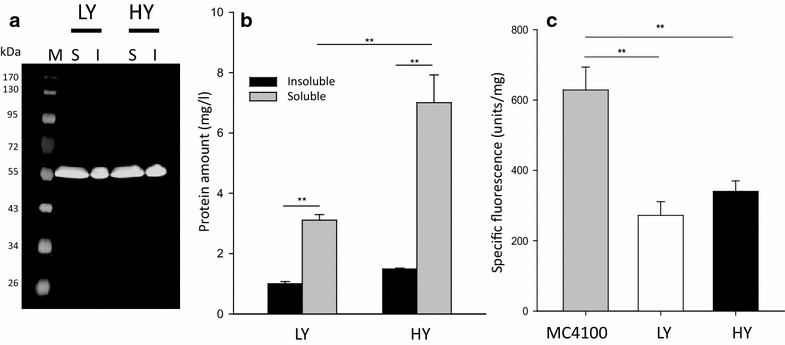
Characterization of VP1GFP produced in *P. pastoris*. **a** Anti-GFP blotting of VP1GFP soluble (S) and insoluble (I) cell fractions: M molecular weight standard (EZ-RUN Pre-Stained Rec Protein Ladder, reference BP3603). **b** Amount of insoluble VP1GFP vs soluble VP1GFP produced in both LY and HY clones. **c** Specific fluorescence of insoluble VP1GFP purified from *P. pastoris* compared with IBs produced in *E. coli* MC4100. (**p < 0.001). Specific fluorescence activity was estimated by the relation between the fluorescence units and the protein amount determined by Western blot

In agreement with light microscopy examination, western blot analysis of cell extracts revealed the occurrence of both soluble and insoluble VP1GFP versions (Fig. 3a), with a higher fraction of soluble protein in both selected clones (Fig. 3b). Both soluble and insoluble VP1GFP versions were fluorescent (Fig. 3a), and the specific fluorescence of insoluble VP1GFP was comparable in HY and LY clones (Fig. 3c), but significantly lower than that shown by VP1GFP IBs produced in the wild type MC4100 *E. coli* strain.

All these data were indicative for the occurrence of insoluble but fluorescent VP1GFP produced in *P. pastoris*, which appeared being organized as protein clusters (Fig. 2b) similar to bacterial IBs. To check for the presence of IB-like material, ultrathin sections of VP1GFP-producing cells were labeled for GFP and examined by TEM.

**Fig. 4:**
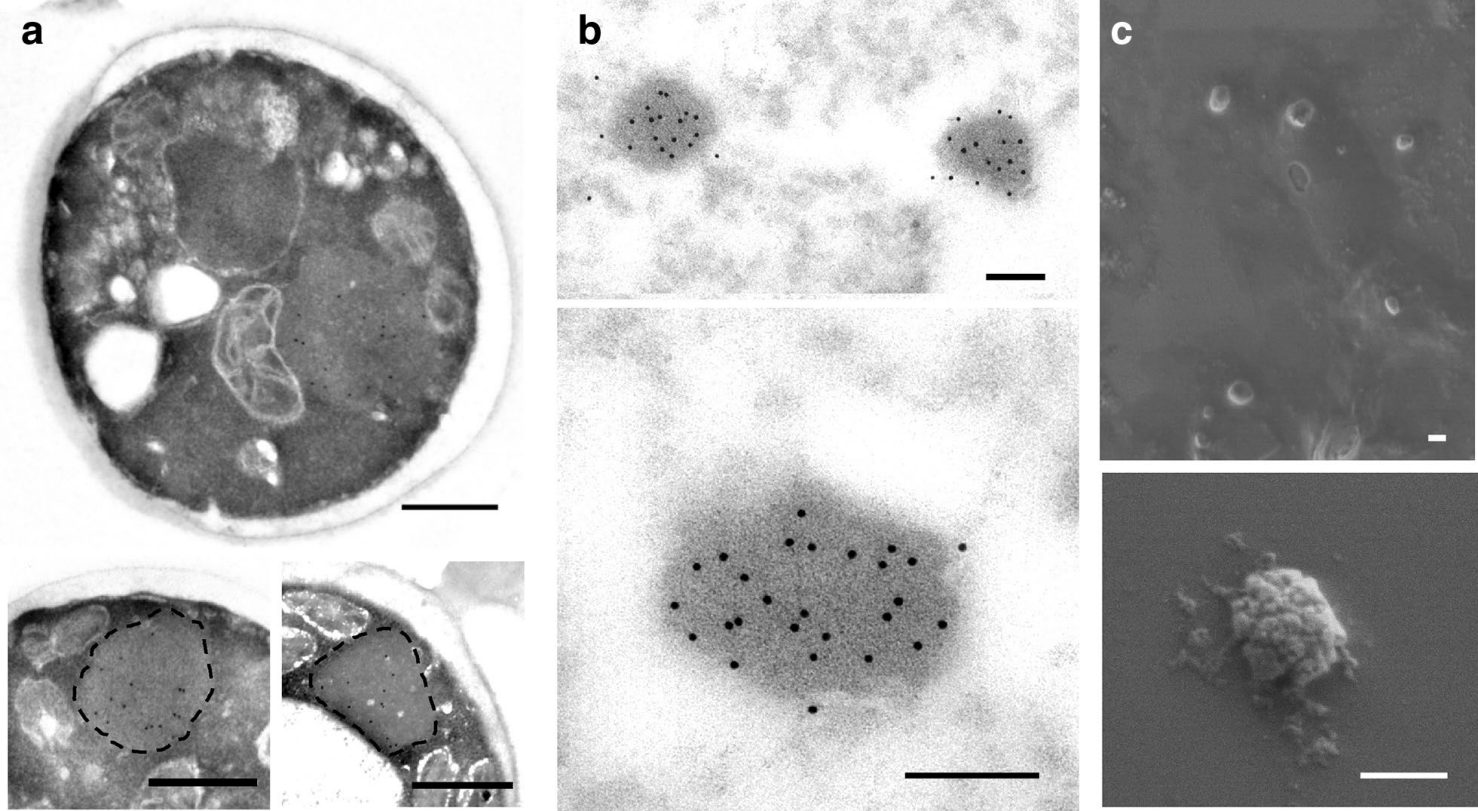
Ultrastructural assessment of VP1GFP IBs in the yeast *Pichia pastoris*. General immuno-TEM view and detail of GFP labeling of entire yeast cells showing well defined intracellular IBs not surrounded by membrane (**a**), and isolated IBs (**b**). Note the heavy immunolocalization revealed by gold particles in both intracellular IBs and purified IBs, demonstrating the significant amounts of GFP in these nanoparticles. Dashed lines are included to stress the IB surfaces. **c** General FESEM view and detail of purified IBs showing their homogenous size, round shape, and rough surface.* Bar* size: **a**, **c** 0.5 μm; **B** 0.2 μm

The obtained images showed the occurrence of GFP-containing protein aggregates, of around 500 nm of size and with regular, pseudo-spherical shape, not surrounded by cell membranes (Fig. 4a–c). These materials showed mechanical stability since they appeared as pseudo-spherical nanoparticles once purified upon cell disruption by harsh mechanical methods (Fig. 4c). The surface of pure inclusions was rough (Fig. 4c), as generically observed in bacterial IBs (including those formed by VP1GFP) [9]. However, surrounding material, probably debris from producing cells, were also observed in some of the particles (Fig. 4c).

**Fig. 5:**
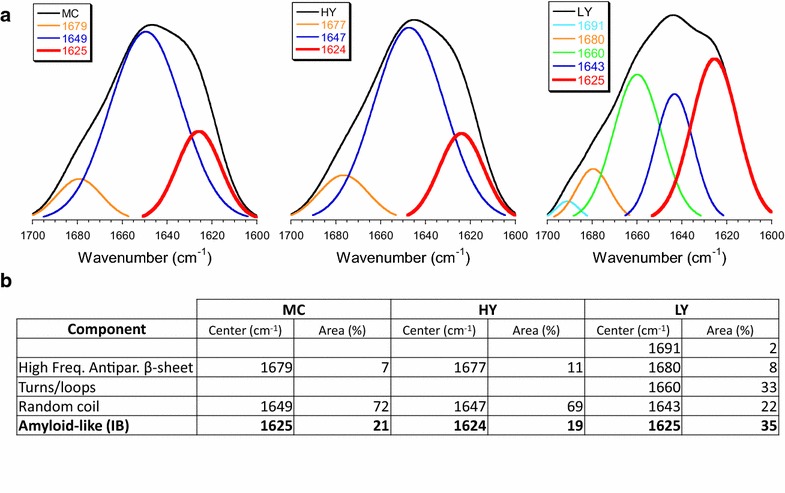
ATR/FTIR spectra of IBs from *E. coli* MC4100 (MC) and *P. pastoris,* in HY and LY strains. **a** Amide I band (*black*) and components resulting from deconvolution. The component featuring IBs is labelled in* red*. **b** Quantitative results of the deconvolution of the amide I spectra. The component featuring IBs is labelled in* bold*

The similarity in morphology and size, aside from the occurrence of fluorescence of VP1GFP IBs produced in *E. coli* and the inclusions formed by the same protein in *P. pastoris* strongly suggested structural and physiological analogies between the protein materials produced in these species, which can be generated efficiently in both bacterial and yeast platforms. Bacterial IBs are characterized by the cross-molecular beta-sheet organization adopted by the forming polypeptide [7], indicative of the occurrence of amyloid-like fibrils [8] that confer porosity and mechanical stability [6]. To explore if the inner molecular architecture was also comparable between both materials we analyzed VP1GFP inclusions by ATR/FTIR (Fig. 5).

There are no differences between amide I band determined in *E. coli* (MC) and HY IBs (Fig. 5a), neither in the result of their deconvolution (Fig. 5b). Both spectra show an amyloid-like (typical IB) component centered at around 1625 cm^−1^ (accompanied by the high frequency antiparallel β-sheet component, centered at around 1680 cm^−1^), and a random coil component at around 1647 cm^−1^, with similar values for the percentage of the corresponding areas. LY, however, is somehow different; the random coil component decreases, the component for turns/loops appears (1660 cm^−1^), and the amyloid-like (IB) component is increased. The band at 1691 cm^−1^ is negligible. The area of the amyloid-like (IB) component is higher for LY IBs (35 vs–20 %, Fig. 5b), but the band is centered at the same wavenumber as for *E. coli* and HY IBs (1625 cm^−1^); therefore, these β-sheets have a similar packing. As a general rule, the position of the β- sheets components shifts to lower wavenumbers as a result of increased hydrogen bonding, flattening of the sheet, or enlarged number of strands [38]. The component centered at 1625 cm^−1^ found for all three cases unequivocally features the typical IB supra-macromolecular architecture. Because LY IBs are expected to be formed at a lower rate than those for HY and *E. coli* materials, it would make sense to attribute the differences found in the LY spectrum to an easier assembling into the IB for the incoming molecules.

**Fig. 6:**
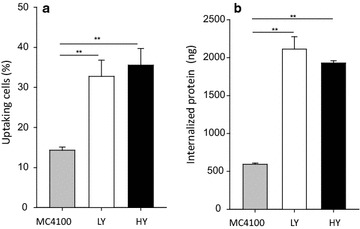
In vitro internalization of VP1GFP IBs produced in *E. coli* (MC4100 strain) and *P. pastoris* (HY and LY clones). **a** Penetration into HeLa cells was measured by flow cytometry counting the percentage of fluorescent cells. **b** Mean fluorescence intensity was measured and the relative amount of VP1GFP into HeLa cells was estimated using the specific fluorescence (FU/µg) of VP1GFP IBs. Row flow cytometry data are shown in the Additional file 3

One of the IB properties that make these protein particles appealing from a biomedical point of view is their membrane-crossing activity [9–11], which allows the penetration of the materials into mammalian cells in absence of toxicity while keeping the functionalities of the IB-forming protein. HeLa cells were exposed to VP1GFP IBs produced in yeast cells, and their potential internalization was monitored by GFP fluorescence retained by cells after a harsh trypsin treatment described to be effective in removing externally attached protein [39]. As observed (Fig. 6), when exposing the same amount of aggregated protein, cells internalized yeast-derived IBs with significantly higher efficiency than bacterial IBs, measured by both percentage of uptaking cells (Fig. 6a) and amount of internalized protein (Fig. 6b).

**Fig. 7:**
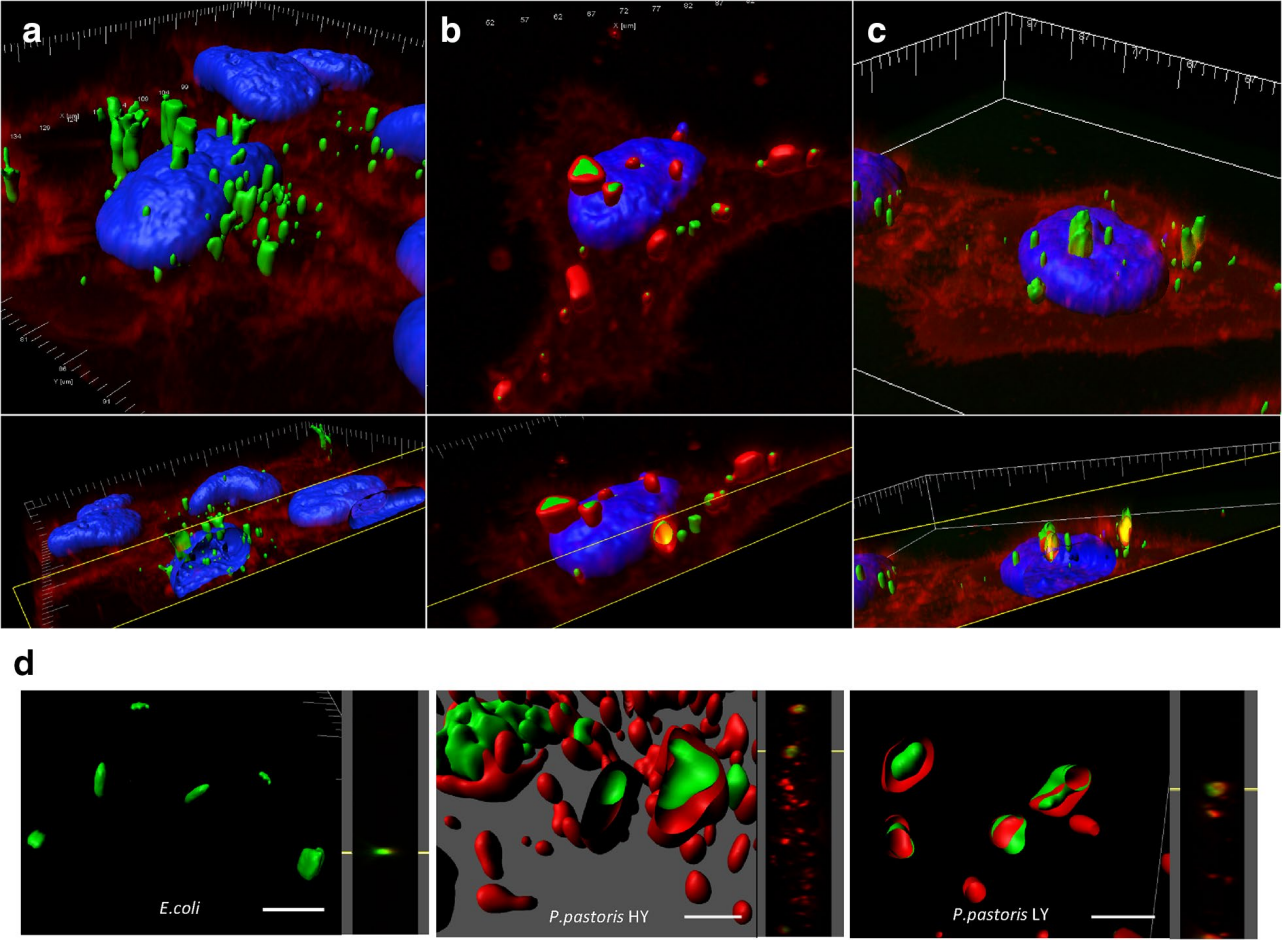
Assessment of cell penetration of VP1GFP IBs exposed to mammalian cells by 3D reconstruction of confocal images. VP1GFP IBs (*green signals*) were produced in **a** *E. coli* MC4100, (**b**) HY clone, and **c** LY clone of *P. pastoris*. Membrane (*red*) and nuclear (*blue*) labelling is apparent. In **d**, three-dimensional reconstruction of confocal microscopy images from VP1GFP IBs produced in either *E.coli* or *P. pastoris.* IBs were stained with cell mask membrane labelling (*red*). Side view signal is displayed next to each image. The* scale bar* represents 500 nm

Intracellular localization of yeast IBs was assessed by confocal microscopy and 3D image reconstruction (Fig. 7a–c), showing in all cases a perinuclear localization. Images were in all cases compatible with macropinocytosis as main uptake mechanism, as recently demonstrated for bacterial IBs with similar size [9]. However, differential membrane staining showing more association of yeast IBs with membranes was also observed (compare Fig. 7a, b), which could be indicative of alternative routes in the penetration of the material. Prior to further verification of uptake routes, and since purified yeast IBs were often observed with associated material (Fig. 4c), we decided first to analyze the potential attachment of yeast cell membranes to the particles. Membrane anchorage to intracellular recombinant proteins is commonly observed [40] and this fact is an intrinsic limitation of this particular cell factory [24]. Indeed, association of membranous structures was observed in purified IBs from yeast but not from bacteria (Fig. 7d), what prompted us to presume that the differential imaging of IB engulfment (Fig. 7a, b) was indeed due to preexisting IB-linked material. The higher association of lipids in yeast IBs might also favor the cellular penetrability of these materials.

## Discussion

The exploration of self-organizing materials fabricated in microbial cells offers appealing tools in different biomedical scenarios [41]. This issue specially applies to self-assembling protein nanomaterials [42]. Resulting from biological fabrication, they recruit regulatable structure and functionality [43, 44] plus an inherent biocompatibility absent in other composites within the nanoscale. The non-toxic nature of most proteins has contributed to the approval of hundreds of protein drugs for medical use with an increasing trend [45], while the potential to engineer protein self-assembling opens a plethora of opportunities for the construction of functional and smart scaffolds in regenerative medicine [46] and in drug delivery [47], among others. While *E. coli* is a choice system for protein production, the removal of contaminant endotoxins from the final product is an increasing matter of concern, as it appears to be a common contaminant of recombinant proteins that might cause, apart from toxicity, miss-consideration of protein activities in different biological assays, both in vitro and in vivo [48–50]. Although endotoxin-free *E. coli* strains have been developed and appear as highly promising for protein production [15, 16], the impact of relevant mutations in these strains on complex protein structures still remains to be fully determined [51]. In fact, features of both the genetic background of the producing strain as well as details of the purification process significantly affect the molecular organization of fine protein oligomers [52, 53].

As an alternative, we explored the potential production of IBs, namely functional protein aggregates of relevant biomedical interest [54], in the yeast *P. pastoris*. The value of IBs is their unusual biophysical properties that involve porosity, mechanical stability, membrane activity, protein functionality and the ability to release active protein upon internalization in mammalian cells [2]. While they are commonly observed in recombinant bacteria and mammalian cells (as functional aggresomes [55, 56]), reports about the formation of IBs or IB-like structures in yeast cells are missing. Results obtained here indicate that IBs are indeed formed in recombinant *P. pastoris* upon the overproduction of an aggregation-prone model protein, under conventional production conditions and without the need to apply stress pressures such as high temperature. IB production appears as not being affected by gene dosage as the protein clusters are formed with comparable efficacies in both HY and LY clones (Fig. 3). However, a slight difference in the inner molecular organization of HY and LY clones (Fig. 5) might be indicative of distinguishable conformational status of VP1GFP, influenced in this case by gene dosage. In this context, the specific fluorescence of VP1GFP yeast IBs is roughly the 50 % of that observed in the equivalent bacterial material (Fig. 3). All these observations, in combination, indicate that despite the different protein quality control network and conformational stress responses acting in yeast and bacteria [57], that might result in less functional IBs in yeast, IB protein in *P. pastoris* can be found in a spectrum of conformational states, as it occurs in *E. coli* [4]. Of course, a significant number of IB-forming proteins should be comparatively examined to determine generic traits of yeasts regarding productivity and functionality of IBs, compared to bacteria.

In any case, yeast IBs are structurally and functionally similar from those produced in *E. coli* (Figs. 2, 4, 5). In fact, these protein clusters, with a size and mechanical stability (even fluorescence intensity) comparable to those from bacteria, (Figs. 2, 3, 6) penetrate cultured mammalian cells very efficiently (Figs. 6, 7), proving their membrane-active properties. The higher penetration of yeast IBs in mammalian cells when compared to bacteria IBs might be in part due to membrane contamination in IB samples, as indicated by associated lipids (Fig. 7) and supported by additional surrounding material seen in electron microscopy (Fig. 4). For many potential applications, the removal of this cellular debris would probably be a critical point in the optimization of IB purification in yeast cells. Contrarily, in bacterial IBs, the separation of whole bacterial cells itself has been an specially complex issue [58].

Finally, the compositional analysis of bacterial IBs is not yet a fully solved issue [59]. IBs are intrinsically complex in composition, as they contain, apart for endotoxins, nucleic acids, lipids and proteins from the producing cell, in proportions that are variable and depending on parameters of the producing process and on the nature of the IB-forming, recombinant polypeptide [1]. Therefore, as their composition might vary from batch to batch, production of these materials in biologically safe microorganisms might smother regulatory constraints even for soft applications such as in cosmetics, food technologies and veterinary medicine, through making the compositional profile less relevant. The food-grade nature of many yeast species [60] as well as their capacity to act as GRAS microorganisms and to produce GRAS products [17, 25] are then additional appealing properties regarding the use of yeasts as IB factories. The recent discovery of IBs in recombinant lactic acid bacteria [61, 62] also fits in this context. In brief, *P. pastoris*, as a representative species of protein-producing yeasts, is revealed here as a suitable potential factory for the production of functional IBs, in absence of potential endotoxin contamination and with the advantages shown by yeast over bacteria regarding, among others, biological safety and post-translational modifications (mainly glycosylation) that are often required for protein functionality.

## Additional files

10.1186/s12934-016-0565-9 VP1GFP gene and protein sequences.
10.1186/s12934-016-0565-9 Raw experimental data.
10.1186/s12934-016-0565-9 Flow cytometry plots.

## Abbreviations

ATR: attenuated total reflectance
DIC: differential interference contrast
FESEM: field emission scanning electron microscope
GAP: glyceraldehyde-3-phosphate dehydrogenase
HY: high yield
IB: inclusion body
LPS: lipopolysaccharide
LY: low yield
LB: lysogeny broth
PMSF: phenylmethylsulfonylfluoride
TEM: transmission electron microscopy
VLP: virus-like particle
