# Supplementary material for: Functional inclusion bodies produced in the yeast Pichia pastoris
Source: Microb Cell Fact. 2016 Oct 1;15:166. doi: 10.1186/s12934-016-0565-9 (PMC5045588; doi:10.1186/s12934-016-0565-9)
Supplement: Supplementary file 1 — 10.1186/s12934-016-0565-9 VP1GFP gene and protein sequences. [file 12934_2016_565_MOESM1_ESM.docx]

**Codon-optimized *VP1GFP* gene**

CAATTGAACAACTATCAAAACACA**ATG**GAAACTACTACTACTGGTGAGTCCGCTGACCCAGTTACTACTACTGTTGAAAACACAATGGAAACTACTACTACTGGTGAGTCCGCTGACCCAGTTACTACTACTGTTGAAAACTACGGTGGTGAGACTCAGGTTCAGAGAAGACACCATACTGACGTTGCTTTCGTTTTGGACTACGGTGGTGAGACTCAGGTTCAGAGAAGACACCATACTGACGTTGCTTTCGTTTTGGACAGATTCGTTGAGGTTACTGTTTCCGACAACCAGCACACTTTGGACGTTATGCAAGCTCACAGATTCGTTGAGGTTACTGTTTCCGACAACCAGCACACTTTGGACGTTATGCAAGCTCACAAGGACAACATCGTTGGTGCTTTGTTGAGAGCTGCTACTTACTACTTCTCCGACTTGGACAAGGACAACATCGTTGGTGCTTTGTTGAGAGCTGCTACTTACTACTTCTCCGACTTGGAGATCGCTGTTACTCACACTGGTAAGTTGACTTGGGTTCCAAACGGTGCTCCAGTTTCCGCGATCGCTGTTACTCACACTGGTAAGTTGACTTGGGTTCCAAACGGTGCTCCAGTTTCCGCTTTGAACAACACTACAAACCCAACTGCTTACCACAAGGGTCCAGTTACTAGATTGGCTTTTTTGAACAACACTACAAACCCAACTGCTTACCACAAGGGTCCAGTTACTAGATTGGCTTTGCCATACACTGCTCCACACAGAGTTTTGGCTACTGCTTACACTGGTACTACTACTTACACGCCATACACTGCTCCACACAGAGTTTTGGCTACTGCTTACACTGGTACTACTACTTACACTGCTTCCGCTAGAGGTGACTCTGCTCACTTGACTACTACTCACGCTAGACACTTGCCAACTGCTTCCGCTAGAGGTGACTCTGCTCACTTGACTACTACTCACGCTAGACACTTGCCAACATCCTTCAACTTCGGTGCTGTTAAGGCTGAGACTATCACTGAGTTGTTGGTTAGAATGAAATCCTTCAACTTCGGTGCTGTTAAGGCTGAGACTATCACTGAGTTGTTGGTTAGAATGAAGAGAGCTGAGTTGTACTGTCCAAGACCAATCTTGCCAATCCAGCCAACTGGTGACAGAAGGAGAGCTGAGTTGTACTGTCCAAGACCAATCTTGCCAATCCAGCCAACTGGTGACAGAAGAAAGCAACAGTTGGTTGCTCCAGCTAAGCAGTTGTTGGGTATTCCATCTAAGGGTGAAGAAAAGCAACAGTTGGTTGCTCCAGCTAAGCAGTTGTTGGGTATTCCATCTAAGGGTGAAGAGTTGTTCACTGGTGTTGTTCCAATCTTGGTTGAGTTGGACGGTGACGTTAACGGTCACAAGTTGTTCACTGGTGTTGTTCCAATCTTGGTTGAGTTGGACGGTGACGTTAACGGTCACAAGTTCTCTGTTTCTGGTGAAGGTGAAGGTGACGCTACTTACGGAAAGTTGACTTTGAAGTTGTTCTCTGTTTCTGGTGAAGGTGAAGGTGACGCTACTTACGGAAAGTTGACTTTGAAGTTCATCTGTACTACTGGAAAGTTGCCAGTTCCATGGCCAACTTTGGTTACTACTTTGACTTACATCTGTACTACTGGAAAGTTGCCAGTTCCATGGCCAACTTTGGTTACTACTTTGACTTACGGTGTTCAGTGTTTCTCCAGATACCCAGACCACATGAAGAGACACGATTTCTTCAAGTCCGGTGTTCAGTGTTTCTCCAGATACCCAGACCACATGAAGAGACACGATTTCTTCAAGTCCGCTATGCCAGAGGGTTACGTTCAAGAGAGAACTATCTCCTTCAAGGACGACGGTAACTACGCTATGCCAGAGGGTTACGTTCAAGAGAGAACTATCTCCTTCAAGGACGACGGTAACTACAAGACTAGAGCTGAGGTTAAGTTCGAGGGTGACACTTTGGTTAACAGAATCGAGTTGAACAAGACTAGAGCTGAGGTTAAGTTCGAGGGTGACACTTTGGTTAACAGAATCGAGTTGAAGGGTATCGACTTCAAAGAGGACGGTAACATCTTGGGTCACAAGTTGGAGTACAACTACAAGGGTATCGACTTCAAAGAGGACGGTAACATCTTGGGTCACAAGTTGGAGTACAACTACAACTCCCACAACGTTTACATCACTGCTGACAAGCAGAAGAACGGTATCAAGGCTAACTTCAACTCCCACAACGTTTACATCACTGCTGACAAGCAGAAGAACGGTATCAAGGCTAACTTCAAGATCAGACACAACATCGAGGACGGTTCCGTTCAGTTGGCTGATCACTACCAACAGAACACGATCAGACACAACATCGAGGACGGTTCCGTTCAGTTGGCTGATCACTACCAACAGAACACCCTATTGGTGACGGTCCAGTTTTGTTGCCAGACAACCACTACTTGTCCACTCAATCCGCTCCTATTGGTGACGGTCCAGTTTTGTTGCCAGACAACCACTACTTGTCCACTCAATCCGCTTTGTCCAAGGACCCAAACGAGAAGAGAGATCACATGGTTTTGTTGGAGTTCGTTACTGCTTTGTCCAAGGACCCAAACGAGAAGAGAGATCACATGGTTTTGTTGGAGTTCGTTACTGCTGCTGGTATCACTCACGGTATGGACGAGTTGTACAAGTAATAGGGCCGCCTCGGCCTGCTGGTATCACTCACGGTATGGACGAGTTGTACAAGTAATAGGGCCGCCTCGGCC

*Mfe*I restriction site

Leader-encoding region

**ATG First VP1GFP-encoding codon**

2x Stop codons

*Sfi*I restriction site

VP1GFP protein

M E T T T T G E S A D P V T T T V E N Y G G E T Q V Q R R H H T D V A F V L D R F V E V T V S D N Q H T L D V M Q A H K D N I V G A L L R A A T Y Y F S D L E I A V T H T G K L T W V P N G A P V S A L N N T T N P T A Y H K G P V T R L A L P Y T A P H R V L A T A Y T G T T T Y T A S A R G D S A H L T T T H A R H L P T S F N F G A V K A E T I T E L L V R M K R A E L Y C P R P I L P I Q P T G D R R K Q Q L V A P A K Q L L G I P S K G E E L F T G V V P I L V E L D G D V N G H K F S V S G E G E G D A T Y G K L T L K F I C T T G K L P V P W P T L V T T L T Y G V Q C F S R Y P D H M K R H D F F K S A M P E G Y V Q E R T I S F K D D G N Y K T R A E V K F E G D T L V N R I E L K G I D F K E D G N I L G H K L E Y N Y N S H N V Y I T A D K Q K N G I K A N F K I R H N I E D G S V Q L A D H Y Q Q N T P I G D G P V L L P D N H Y L S T Q S A L S K D P N E K R D H M V L L E F V T A A G I T H G M D E L Y K
