# Supplementary material for: Functional inclusion bodies produced in the yeast Pichia pastoris
Source: Microb Cell Fact. 2016 Oct 1;15:166. doi: 10.1186/s12934-016-0565-9 (PMC5045588; doi:10.1186/s12934-016-0565-9)
Supplement: Supplementary file 3 — 10.1186/s12934-016-0565-9 Flow cytometry plots. [file 12934_2016_565_MOESM3_ESM.docx]

**
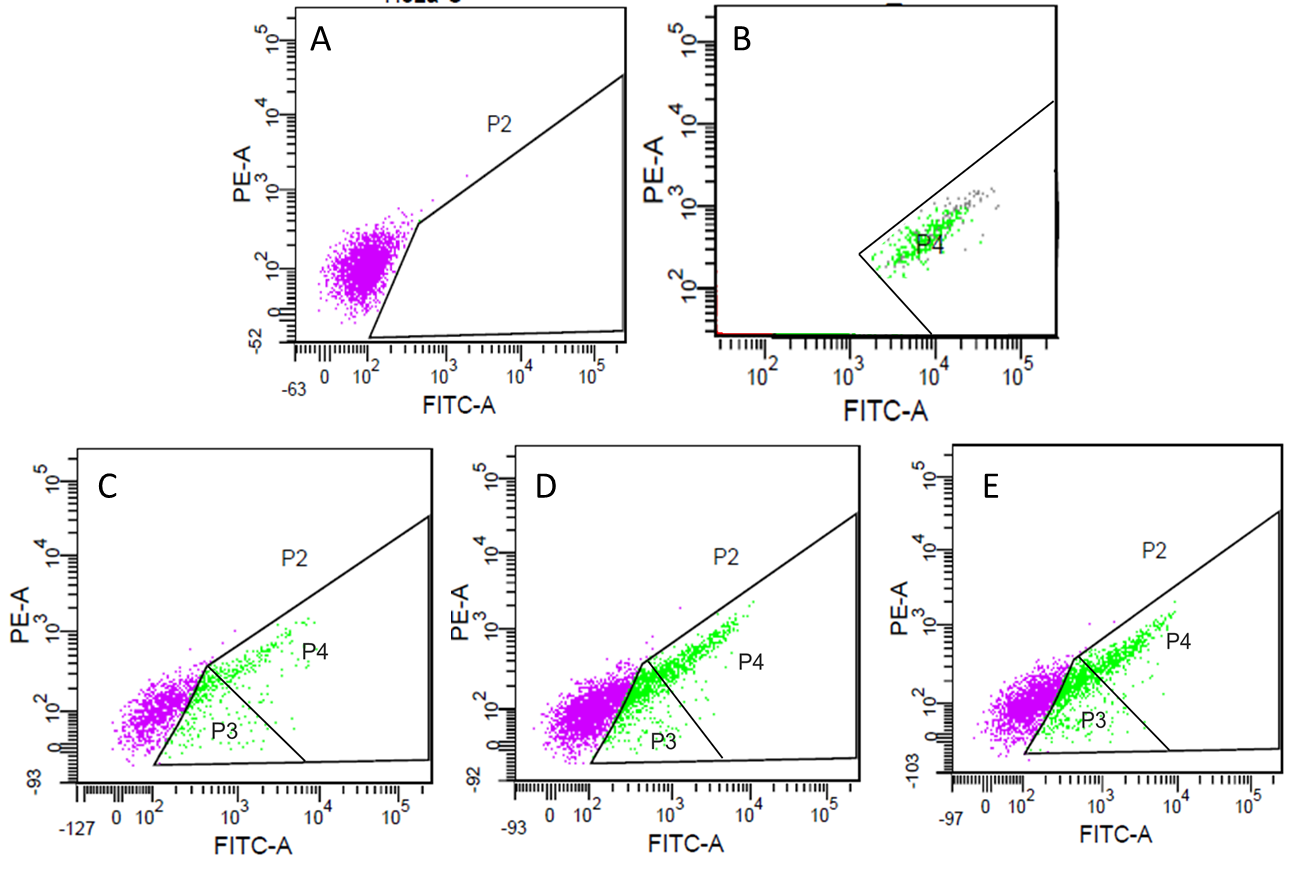
**

**Distribution of cellular population determined by FACS. (A) HeLa cells incubated without IBs (negative control) are represented by P2 population, and (B) purified VP1GFP IBs in absence of cells are delimited by P4 population. Uptaking HeLa cells are represented by P3 population, upon incubation with VP1GFP IBs produced in *E. coli* (C), *P. pastoris* LY (D) or *P. pastoris* HY (E).**
